# Annealing-Induced Changes in the Nature of Point Defects in Sublimation-Grown Cubic Silicon Carbide

**DOI:** 10.3390/ma12152487

**Published:** 2019-08-06

**Authors:** Michael Schöler, Clemens Brecht, Peter J. Wellmann

**Affiliations:** Crystal Growth Lab, Materials Department 6 (i-meet), Friedrich-Alexander University Erlangen-Nürnberg (FAU), Martensstr. 7, D-91058 Erlangen, Germany

**Keywords:** 3C-SiC, sublimation growth, doping, point defects, defect engineering, photoluminescence

## Abstract

In recent years, cubic silicon carbide (3C-SiC) has gained increasing interest as semiconductor material for energy saving and optoelectronic applications, such as intermediate-band solar cells, photoelectrochemical water splitting, and quantum key distribution, just to name a few. All these applications critically depend on further understanding of defect behavior at the atomic level and the possibility to actively control distinct defects. In this work, dopants as well as intrinsic defects were introduced into the 3C-SiC material in situ during sublimation growth. A series of isochronal temperature treatments were performed in order to investigate the temperature-dependent annealing behavior of point defects. The material was analyzed by temperature-dependent photoluminescence (PL) measurements. In our study, we found a variation in the overall PL intensity which can be considered as an indication of annealing-induced changes in structure, composition or concentration of point defects. Moreover, a number of dopant-related as well as intrinsic defects were identified. Among these defects, there were strong indications for the presence of the negatively charged nitrogen vacancy complex (N_C_–V_Si_)^−^, which is considered a promising candidate for spin qubits.

## 1. Introduction

Over the last decades, silicon carbide (SiC) has been established as a promising material for various applications due to the fact of its outstanding physical, electrical, and optical properties. A wide band gap, high break-down field strength, high-saturation drift velocity, and high thermal conductivity fostered applications for high-power and high-temperature electronics [1,2,3]. Radiation hardness and chemical inertness makes SiC promising for sensing and detectors [4,5,6]. Due to the facts of its biocompatibility, SiC is used for various biomedical applications such as coatings and sensors [7,8]. In recent years, SiC is also gaining increasing interest as material for quantum applications. Deep level defects in SiC can be suitable for spin-qubits and single-photon-sources (SPS) which are the basic unit for quantum key distribution (QKD) networks [9], which, with SPS, allows inherently secure data communication by encrypting information which can considerably influence future communication. 

For the latter application, the cubic modification of silicon carbide (3C-SiC) is a promising candidate as it provides higher symmetry of the crystal lattice in comparison with its hexagonal counterparts [10]. Doping of 3C-SiC opens up further opportunities. Boron-doped 3C-SiC may act as an ideal material for intermediate-band solar cells due to the almost perfect deep B-level within the band gap [11]. Aluminum-doped 3C-SiC could lead to the development of efficient photoelectrochemical water splitting cells for hydrogen generation [12,13]. Therefore, deep knowledge about optical properties of 3C-SiC, especially with regard to dopants and point defects, is essential for future applications.

In this work, we have prepared freestanding 3C-SiC bulk material which is co-doped with nitrogen, boron, and aluminum. Temperature-dependent photoluminescence (PL) measurements were performed in order to characterize the electronic levels from dopants and intrinsic defects. Peaks were identified and assigned with data from the literature and in consideration of growth conditions. By performing isochronal annealing of the 3C-SiC sample, temperature-dependent changes of the PL signal were used to interpret alteration of defects within the bulk material. In contrast to most other works in this field, the defects were incorporated in situ during sublimation growth and not by implantation or irradiation.

## 2. Materials and Methods 

A sample (S) of freestanding, single crystalline 3C-SiC was grown by epitaxial sublimation growth (ESG). The homoepitaxial growth by ESG was performed on a (100)-oriented, 4° off-axis 3C-SiC seed-layer which was previously grown by chemical vapor deposition (CVD) on a silicon substrate. A transfer-process developed in our lab was used to transfer this 3C-SiC seed to a polycrystalline SiC carrier for mechanical stabilization [14,15]. The resulting seed-stack was used in the ESG growth cell for subsequent growth of high-quality bulk-like 3C-SiC. For stabilization of the cubic polytype, growth was performed under Si-rich gas phase conditions and high supersaturation of the Si-containing gas species [16]. These requirements were achieved by using a small distance between the source material and seed-stack, resulting in high temperature gradients and the use of a tantalum foil in order to getter carbon from the gas phase. A polycrystalline SiC bulk wafer prepared in our own lab by physical vapor transport (PVT) method was used as source material for the growth and the doping. In ESG, doping type and doping level can be varied by the choice of the doping of the poly-SiC source material. Depending on the element, the dopant concentration of the source material is approximately reproduced in the growing 3C-SiC layer. In order to enable the formation of various dopant-related point-defects and defect complexes, a poly-SiC wafer doped with nitrogen, boron, and aluminum was chosen as source material for the sample investigated in this study. From the secondary ion mass spectrometry (SIMS) measurements, followed co-doping of the final 3C-SiC layer. The concentrations were determined to 6.58 × 10^20^ cm^−3^, 1.44 × 10^17^ cm^−3^, and 2.56 × 10^18^ cm^−3^ for N, B, and Al, respectively. Dopant concentrations were calculated using reference samples with well-known concentrations of the elements therein. The dimension of the (100)-oriented 4° off-axis layer was (25 × 25) mm^2^. 

Specific attention should be drawn to the processing of the sample. During growth by ESG, the 3C-SiC seed and the growing layer were mounted to a polycrystalline SiC-carrier wafer, which partially sublimes during a standard growth run. In order to fully eliminate the carrier and to get a freestanding 3C-SiC layer with maximum thickness, the growth process was extended beyond the standard process duration. By doing so, the source material was also completely consumed. This procedure allowed the elimination of any post-processing of the sample which would usually contain temperature treatments that could unintentionally influence the point defects in the material. The 3C-SiC sample analyzed in this work had a thickness of 755 ± 11 µm and was grown with a growth rate of approximately 168 ± 2 µm/h at a growth temperature of 1901 ± 3 °C.

**Figure 1:**
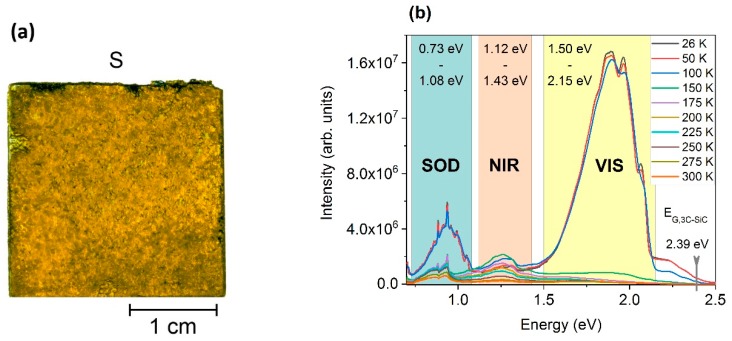
As-grown bulk cubic silicon carbide (3C-SiC) sample and typical temperature dependent photoluminescence (PL) spectra of the sample: (**a**) backlight image of the as-grown bulk 3C-SiC sample prepared by epitaxial sublimation growth. The inhomogeneities of the coloring originate from carbon inclusions, protrusions, and, primarily, residuals from the seed mounting on the back side of the layer; (**b**) typical temperature-dependent PL-spectra of the as-grown 3C-SiC. The spectra were acquired with a 405 nm laser. Three sections were classified and labeled as second-order-diffraction (SOD), near-infrared (NIR), and visible (VIS) luminescence.

An 8 × 9 mm^2^ piece of the sample depicted in Figure 1a was used to perform isochronal annealing from 100 °C to 1300 °C with a step size of 150 °C in order to evaluate the influence of thermal treatments on point defects in the material. After each temperature step, the sample was analyzed by photoluminescence (PL). Temperature treatments were carried out in a tube furnace GERO-RO (Carbolite Gero, Neuhausen, GERMANY) under nitrogen atmosphere. A rate of 20 K/min was used for ramp up to the envisaged temperature. After a hold time of 30 min at elevated temperature, cool down was performed with an initial rate of approximately 73 K/min by switching of the furnace. 

Temperature dependent photoluminescence measurements were performed between 26 K and 300 K with a step size of 25 K using a CTI-Cryogenics (Helix Technology Corp., Mansfield, MA, USA) closed-cycle-He-cryostat in combination with a temperature controller LakeShore 330 (Lake Shore Cryogenics Inc., Westerville, OH, USA). A laser diode with 405 nm (CUBE 405-100C, Coherent, Wilsonville, OR, USA) in combination with a 405 nm band pass as well as a 450 nm long pass filter were used for above band gap excitation. All measurements were conducted with 50 mW laser power and a spot size of approximately 480 µm in diameter. The penetration depth of the laser was approximately 10 µm in the case of high-quality 3C-SiC [17]. The spectra were acquired between 450 nm and 1700 nm with a cooled InGaAs array detector (Symphony IGA-512×1, Horiba, Edison, NJ, USA) and a cooled charge-coupled device (CCD) detector (CCD-1024×128-6, Horiba, Edison, NJ, USA) utilizing a monochromator Horiba TRIAX 552 (grating: 150 mm^−1^). All spectra were converted to energy-scale by applying Jacobian conversion [18,19].

## 3. Results

As shown in previous works [20,21], as-grown bulk 3C-SiC exhibits typical temperature dependent PL-spectra, as can be seen in Figure 1b. At low temperatures, a bright luminescence in the visible range (VIS) can be observed. Related to this, a remarkable second-order-diffraction (SOD) of the VIS can be observed with an overall behavior equal to its visible origin. The third area in Figure 1b is referred to as near-infrared (NIR). The origin of the luminescence in this area was strongly dependent on growth-rate and was assigned to C-related clusters of defects [20]. In fact, the area denoted as SOD is the superimposition of second-order-diffraction of VIS and the luminescence from distinct point defects in the near-infrared. From a physical perspective, the luminance of both, SOD and NIR, lie within the range of near-infrared. However, for better clarity, the classification of SOD for second-order-diffraction dominated luminescence and NIR for solely defect-based infrared-related luminescence seems reasonable.

**Figure 2:**
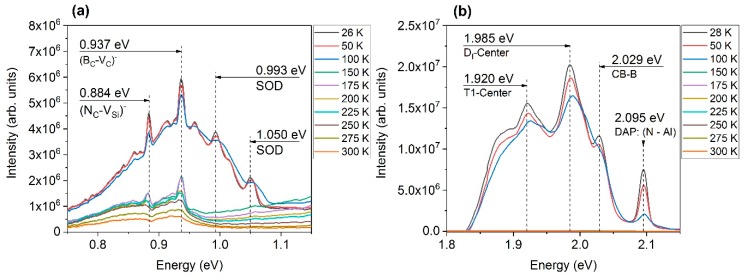
Identification of peak positions in the second-order-diffraction (SOD) and visible (VIS) regimes: (**a**) Within the SOD regime, four distinct peaks can be identified. Two of them were assigned to SOD of VIS and two originated from point defects. The spectra were acquired with the 405 nm laser and the InGaAs detector. (**b**) Within the VIS regime, four distinct peaks can be identified. Two of them were assigned to donor–acceptor pairs (DAP) and two of them were assigned to defect centers originating from point defects. The spectra were acquired with the 405 nm laser and the charge-coupled device (CCD) detector after the annealing step at 100 °C.

For low-temperature PL, there was strong luminescence of VIS and SOD. For PL-temperatures equal or higher than 150 K, the intensity of VIS dropped to a low value. A similar behavior can be observed for SOD, indicating the joint origin of the luminescence. However, the SOD consisted of the superimposition of second-order-diffraction and luminescence from distinct defects. While the SOD peaks at 0.993 eV and 1.050 eV (see Figure 2a) dropped in the same manner as VIS, some discrete peaks remained visible up to room temperature. Two peaks at 0.937 eV and 0.884 eV could be identified. In Figure 2b, the upper area of the VIS regime is displayed. Four discrete peaks can be identified at positions of 1.920 eV, 1.985 eV, 2.029 eV, and 2.095 eV. Whereas the first two originate from point defects, defect complexes, and structural defects, the second two can be assigned to donor–acceptor pair (DAP) related transitions of dopants.

**Figure 3:**
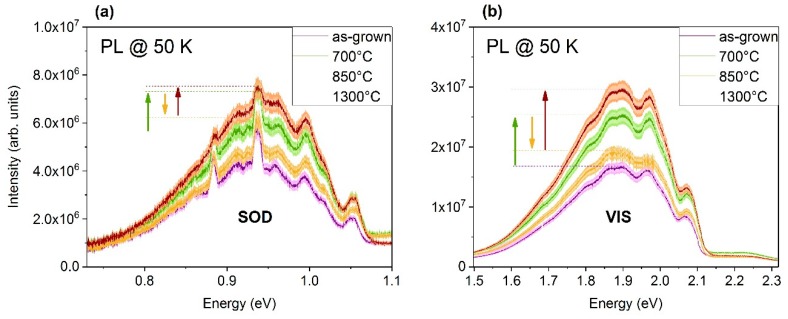
Behavior of (**a**) SOD and (**b**) VIS during temperature treatments. For improved clarity, only distinct temperatures of “as-grown” (without T-treatment), 700 °C, 850 °C, and 1300 °C are shown. The arrows indicate the shift of PL spectra after annealing. All spectra in both diagrams were acquired at a PL temperature of 50 K after temperature treatment. For excitation, the 405 nm laser was used in combination with the InGaAs detector. The shaded areas describe the variance of the reproducible reaching of the measuring position.

An 8 × 9 mm^2^ piece of the sample (S) shown in Figure 1a was used to perform a series of isochronal temperature treatments. Figure 3 displays a selection of distinct annealing temperatures where significant changes in the intensity of the PL-spectra can be observed. Both areas, SOD and VIS, show the same behavior. The as-grown sample exhibited the lowest intensity. When the sample was annealed, the luminescence started to rise until an annealing temperature of 700 °C was reached (green arrow). After the temperature treatment at 850 °C, the luminescence dropped again (yellow arrow). Annealing at a temperature of 1300 °C increased the intensity again and led to the highest intensity measured (red arrow). 

**Figure 4:**
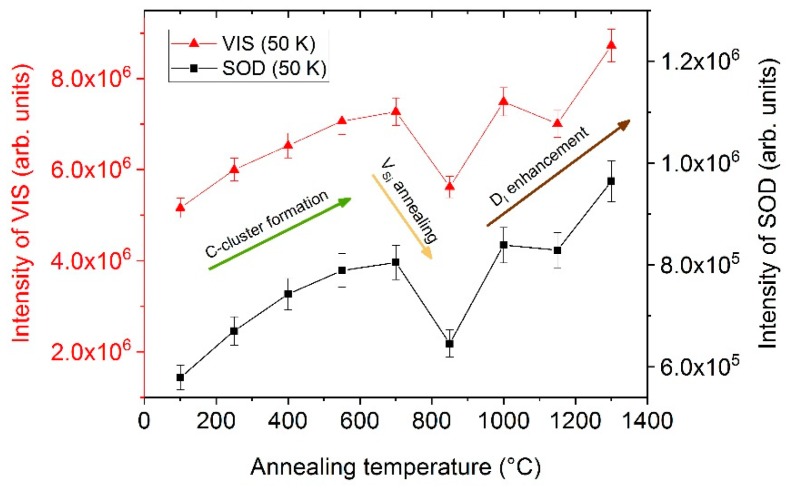
Integrated intensities of the VIS regime (1.5–2.15 eV) and SOD regime (0.73–1.08 eV) as a function of annealing temperature. Mechanisms explaining the curve characteristics are indicated in the diagram. The spectra were acquired with the 405 nm laser and the InGaAs detector at a measuring temperature of 50 K.

A more detailed presentation of the corresponding results is given in the diagram in Figure 4. The graph shows the integrated intensities of the VIS and SOD regimes for each annealing step. The integration was performed from 0.73 eV to 1.08 eV and from 1.5 eV to 2.15 eV for SOD and VIS, respectively. From Figure 4 it is apparent that the annealing at 850 °C led to a drop of luminescence which can be almost reproduced by the subsequent temperature treatment at 1000 °C. A second smaller drop in intensity can be observed for the annealing at 1050 °C. The overall trend indicates an increasing luminescence intensity with increasing annealing temperatures up to 1300 °C.

## 4. Discussion

The assignment of peaks was made by comparing the experimentally obtained peak positions with values from the literature taking into account the process and growth conditions during sample preparation. In the VIS regime, peaks with center wavelengths at 1.920 eV, 1.985 eV, 2.029 eV, and 2.095 eV were identified (see Figure 2b). Since the intensity of the whole band significantly dropped during heating up from low temperature PL and exceeding 150 K, the origin of the band could lie in defects that will be ionized through the gain of additional thermal energy. Therefore, donor–acceptor pair (DAP) transitions could be responsible for the peaks. Assuming energy levels of 0.0565 eV [22] and 0.254 eV [22] with respect to the energy gap of 3C-SiC (2.39 eV, [23]) for nitrogen and aluminum, respectively, the resulting N–Al transition energy of 2.080 eV may be assigned to the peak at 2.095 eV. With increased doping levels, the ionization energies of the dopants shrink. The same holds for a high charge carrier concentration that lowers the bandgap energy. Assuming the presence of the shallow boron level at 0.35 eV [24], various possibilities exist for the origin of the peak at 2.029 eV. A transition from the conduction band (CB) to the B-level would result in an energy of 2.040 eV, whereas for the DAP: N–B would give luminous response with an energy of 1.984 eV. Moreover, Al–B complexes could be involved due to the doping of the sample. However, an unambiguous assignment was not possible.

For the peak at 1.920 eV, data from the literature offers several possible origins. Choyke et al. [25] reported the G-band in 3C-SiC with a center wavelength of 1.912 eV which could correspond with the peak at 1.920 eV. Choyke et al. also reported side bands G1 and G2 at 1.832 eV and 1.796 eV, respectively. These bands were not observed in the present analysis. However, the side bands could lie underneath the superimposition of broad bands at the low-energy flank of VIS. The origin of the G-band was assigned to dislocations and structural defects [25]. Another defect center is the T1 center [26], which was observed for 3C-SiC grown by chemical vapor deposition. Its position at 1.913 eV [27] was assigned to originate from silicon vacancies (V_Si_) [26]. The long growth time of the sample with a full consumption of the source material may have caused a change in the gas-phase composition towards the end of the process. This might be due to the degradation of the tantalum used as carbon-getter (see Reference [14]) or an out-diffusion of silicon due to the full consumption of the source material. Therefore, the presence of silicon vacancies seems plausible. The third possible origin of the peak at 1.920 eV is referred to as δ-center [27]. This center was determined to lie at 1.922 eV [27], which is in good agreement with the value of 1.920 eV. Itoh et al. [27] observed the δ-center only in irradiated material and assigned the defect to radiation-induced defects. A typical behavior of the δ-center is an increasing intensity up to 50 K. Due to the limited set of PL temperatures, this effect was not investigated and remains an open issue for future analysis.

Itoh et al. [27] reported a sharp line at 1.973 eV and a broad band at 1.92 eV, which were associated with the D_I_-line and the G-band, as described by Choyke et al. [25]. The band should be apparent for PL temperatures up to 100 K and disappears for higher temperatures. This would be roughly in accordance with the results presented in this work. The position of the D_I_-center lies near the position of the 1.985 eV peak. The origin of the D_I_-center was first assigned to a di-vacancy complex (V_C_–V_Si_) but later correlated with an antisite-complex (Si_C_–C_Si_), an isolated silicon antisite (Si_C_) or small clusters of silicon and carbon (Si_i_–C_i_) [28,29,30]. Another possible origin is the DAP: N–B (shallow) transition which would result in an energy of 1.984 eV, as described above.

In the SOD regime (see Figure 2a), peaks with center wavelengths at 0.884 eV, 0.937 eV, 0.993 eV, and 1.050 eV were identified. As the peaks at 0.993 eV and 1.050 eV showed the same temperature-dependent behavior as the VIS band and disappear for PL temperatures higher than 100 K, they were identified as second-order-diffraction of VIS. Within the measurement error, their energetic positions were roughly half of the corresponding peaks at 1.985 eV (for 0.993 eV) and 2.095 eV (for 1.050 eV) in the VIS regime. In contrast, the other peaks in the SOD regime remained apparent at least up to 225 K during PL characterization. The origin of the 0.937 eV peak could not be assigned without doubt. However, a boron-related defect might be responsible for the luminescence. Boron tends to form various complexes with intrinsic defects. These structures give rise to numerous intermediate levels in this area of the band gap. The (B_C_–V_C_)^–^ complex, for example, gives rise to a level at 1.4 eV [31]. Due to the doping and the low formation energies of this complex, its presence seems plausible. A transition from the conduction band to the B-complex would result in an energy of 0.98 eV, which is close to the observed peak positon. The peak at 0.884 eV is in good agreement with the ionization level of the (N_C_–V_Si_)^−^ defect, which was theoretically determined to lie at 0.87 eV [32] or 0.89 eV [10]. It should be noted that this defect was not observed in samples previously grown with our setup. However, the growth conditions of the presented sample vary from our standard process. The pressure during growth was higher than usual which will result in an increased incorporation of nitrogen. Additionally, the extended growth conditions could have led to a lack of silicon containing gas species at least during the last period of growth. Therefore, the presence of both high concentrations of N and silicon vacancies (V_Si_) corresponds with expectations. High concentrations of these defects cause many defects close to each other which can merge and generate complexes by reducing their total energy. Various paths for the formation of the (N_C_–V_Si_)^−^ defect might be possible. First, with a barrier of 3.5 eV, the transition of (N_C_–V_Si_)^−^ from V_Si_ and N_C_ can occur. The energetic benefit would be 2 eV [28]. At high temperatures of 1900 °C, the migration of vacancies and, therefore, the described transition should be possible during growth. Second, if the necessary defects already exist within the material, a merger of (NC)_C_ and V_C_–V_Si_ to form N_C_–V_Si_ might be favorable. The barrier for this process should be 0.2 eV and the energy gain of the transition would be 7.4 eV [28]. As (NC)_C_ is the standard configuration of N interstitials and di-vacancies are frequently occurring intrinsic defects, the last mechanism might be preferred from an energetic point of view.

From Figure 3, it follows that peaks neither appear nor disappear during temperature treatments between 100 °C and 1300 °C. However, the overall intensities of the luminescence in the VIS and SOD regimes exhibit a variation depending on annealing temperature. Hence, even if there was no assignable peak–defect pair generated or annihilated, the presented results provide reasonable indications for temperature-dependent changes in the composition and concentration of point defects in bulk 3C-SiC. As described in the previous section, the majority of the intensity in the SOD band was related to second-order-diffraction of the VIS regime. Therefore, the temperature-dependent changes in the SOD luminescence can be considered mainly as an artefact of the VIS band accordingly with the almost identical behavior of both.

In Figure 4, the integrated peak intensities of VIS (1.5–2.15 eV) and SOD (0.73–1.08 eV) are presented versus annealing temperature. The intensities were calculated from the PL spectra acquired at 50 K. With increasing annealing temperature, the intensity increased as well with a major drop after the annealing at 850 °C and a minor drop after the temperature treatment at around 1050 °C. Such changes in PL signal intensity can generally be explained by either alterations in the number of point defects or in the number of recombination centers [29]. As the changes in intensity cannot be attributed to single peaks or defects, the observed effects were not assigned to a change in the number of distinct point defects, but to a change in the concentration of non-radiative transitions.

Clusters of carbon atoms frequently emerge in SiC and can exist in a variety of different configurations [33,34,35,36]. Each configuration introduces electronic levels within the band gap. A high number of C-clusters close to each other can, therefore, open up paths for recombination of charge carriers. From theoretical considerations it follows that the migration of most common C-interstitials C_sp<100>_ and C_spSi<100>_ occurs in the energy range up to 0.7 eV with charge states of 0 and 1 [37]. Annealing at temperatures up to 700 °C should already provide enough energy to thermally activate migration. A high number of C-interstitials in combination with low formation and migration energies could lead to the aggregation of C-atoms. Due to the variety of configurations, the aggregates can influence wide ranges of the PL spectrum. Even if the details and mechanisms are not completely clear, the increase in luminescence up to annealing temperatures of 700 °C is assigned to C-cluster formation.

Freitas et al. [38] observed an increasing intensity of the D_I_-center up to annealing temperatures of 1600 °C, which supports the assignment of the 1.985 eV peak. Lefèvre et al. [29] observed an increase in the D_I_-center, too. However, the increase started only for annealing temperatures equal or higher than 1100 K. The increasing intensity for annealing temperatures higher than 850 °C may, therefore, be assigned to the enhancement of the D_I_-center.

The drop in intensity around 850 °C might be related to silicon vacancies (T1 center). Itoh et al. [39] conducted electron-spin-resonance analysis and found three stages for the annealing of V_Si_^−^. The stages were located at 150 °C, 350 °C, and 750 °C with the last being the most pronounced. Due to the expected high concentration of V_Si_ within the material and the good agreement between the reported third stage and the results in this work, the drop at 850 °C was assigned to V_Si_^−^ annealing.

In this work, a variation in the overall PL intensity could be observed which can be considered an indication for annealing-induced changes in structure or concentration of point defects. However, this effect could not be assigned to a distinct defect or complex which was generated or completely annihilated during temperature treatment. This may be related to the method of introducing the defects into the material. In the literature, most studies report on the theoretical considerations or point defects generated by irradiation. The latter will lead to a situation which is different from in situ generation of point defects during growth by sublimation. Especially, concentration, distance, and configuration of defects will be influenced by the method of defect generation. The findings in this work are essential when it comes to defect engineering for technical utilization of point defects in 3C-SiC.

## 5. Conclusions

The peak at 2.095 eV was assigned to the DAP transition of N–Al, whereas the peak at 2.029 eV could result from CB-B or DAP: N–B transitions. The origin of the peak at 1.920 eV could not be determined unambiguously. However, an association with the T1-center and, therefore, a connection with silicon vacancies seems reasonable due to the growth conditions. A connection with the D_I_-center was found for the 1.985 eV peak which originated from an intrinsic defect complex.

Presumably, due to the extended growth conditions, a peak with a center wavelength of 0.884 eV was observed. The transition is in good agreement with the (N_C_–V_Si_)^−^ defect which is a promising candidate for qubits.

Isochronal temperature treatments between 100 °C and 1300 °C revealed changes in the number and character of radiative defects depending on annealing temperature. Up to 700 °C, an increasing PL intensity was observed, which was assigned to the broad influence of aggregates from carbon interstitials. A drop in the luminescence at approximately 850 °C can be explained by annealing mechanisms of silicon vacancies. The subsequent increase in the PL intensity is explained by the enhancement of the D_I_-center. We assumed that temperature treatments did not lead to the complete elimination of defects but rather to a change in the structure, composition or concentration of defects.
